# Effects of a multimodal exercise intervention on physical and cognitive functions in patients with chronic low back pain (MultiMove): study protocol for a randomized controlled trial

**DOI:** 10.1186/s12877-021-02093-1

**Published:** 2021-03-02

**Authors:** Lutz Schega, Britta Kaps, Kim-Charline Broscheid, Robert Bielitzki, Martin Behrens, Katharina Meiler, Steffen Drange, Jörg Franke

**Affiliations:** 1 Health and Physical Activity, Department of Sport Science, Institute III, Otto von Guericke University Magdeburg, Zschokkestraße 32, 39104 Magdeburg, Germany; 2 Department of Orthopaedic Surgery, Klinikum Magdeburg gGmbH, Birkenallee 34, 39130 Magdeburg, Germany

**Keywords:** Motor control, Dance, Gait variability, Dual task, Functional near-infrared spectroscopy

## Abstract

**Background:**

Chronic low back pain (CLBP) is a common medical condition in adults over the age of 50. It is associated with severe disability, ranging from physical impairments to psychosocial distress. Since current treatments provide only small to moderate short-term effects, alternative interventions are required, whereby guidelines recommended multimodal approaches. Dancing can be considered as an inherently multimodal approach, as it requires a combination of physical and cognitive functions. Furthermore, it has already been applied effectively in neurorehabilitation. Therefore, it seems promising to merge a dance-therapeutic component together with motor-cognitive, strength and flexibility exercises in a novel multimodal treatment (MultiMove) to target the impaired everyday mobility and cognition of CLBP patients. The aim of this study is to analyse specific physical, cognitive and psychosocial effects of MultiMove in CLBP patients.

**Methods:**

A prospective, two-arm, single-blinded, randomized controlled trial will be conducted with an estimated sample size of 100 CLBP patients, assigned to either the MultiMove group or a control group. The intervention group will receive MultiMove twice a week for 60 min each over a period of 12 weeks. The primary outcome will be the mobility and function of the lower extremities assessed by the Timed Up-and-Go Test. Secondary outcomes comprise further physical and physiological functions (e.g. gait variability and haemodynamic response in the prefrontal cortex during motor-cognitive dual tasks), subjective health state (e.g. disability in daily life), executive functions (e.g. cognitive flexibility) and psychosocial aspects (e.g. kinesiophobia). Measures will be taken at baseline, after the intervention and at a 12-week follow-up. It is assumed that MultiMove improves the mentioned outcome parameters.

**Discussion:**

The combined assessment of changes in physical and cognitive functions as well as neuropsychological aspects in response to MultiMove will allow a better understanding of the motor-cognitive adaptations induced by multimodal exercises in CLBP patients. The specific conclusions will lead to recommendations for the conservative treatment approach in this clinically relevant patient group.

**Trial registration:**

German Clinical Trial Register (ID: DRKS00021696 / 10.07.2020), https://www.drks.de/drks_web/navigate.do?navigationId=trial.HTML&TRIAL_ID=DRKS00021696

## Background

Low back pain (LBP) is a frequent medical condition and major economic health issue in Germany with a lifetime prevalence of around 85% in the population [1]. In a study considering the global burden of diseases, LBP was classified as the condition highest in terms of disability. Furthermore, it was shown that the prevalence increases with age and that the chronic type of LBP (CLBP, with and without leg pain) was associated with the most severe disability [2]. Thereby, CLBP is defined as pain in the lumbar region of the spine lasting for at least 3 months [3]. Although, only a small percentage of LBP patients develop CLBP [3, 4], they are responsible for the majority of costs associated with LBP caused by recurrent health care consultations, occupational incapacity and early retirement [5–8]. Degenerative changes in the lumbar spine are strongly associated with CLBP [5, 9]. However, in most of the cases they cannot sufficiently explain the underlying pain mechanisms and the related impairments [4, 10–12].

On a physical level, CLBP patients can experience limitations such as insufficient muscular trunk stabilisation and strength [13], as well as a poor static postural control and an altered gait performance [14–17]. Moreover, research indicates that motor-cognitive dual task performance (e.g. walking while performing a cognitive task) is reduced in CLBP patients [14, 18]. This could be explained by the fact that pain can interfere with gait control, e.g. executive functioning [14, 19]. This is consistent with the observation that patients with chronic pain showed reduced cognitive functioning (e.g. executive functions) [20]. These motor-cognitive deficits lead to limitations in daily activities and may provoke an increased risk of falling [13, 19, 21]. Nevertheless, it is essential to consider that also psychological factors like anxiety, depression and/or passive coping strategies affect the development and maintenance of CLBP [4, 22]. In addition, these impairments reinforce each other: e.g. the fear of pain leads to a decreased daily activity level (described as kinesiophobia [23]), contributing to the insufficient muscular stabilisation and disability level [16, 24]. These multidimensional negative experiences impair the patients beyond the chronic pain and contribute to a reduction in health-related quality of life [25, 26].

To address the multifaceted impairments of CLBP, multidisciplinary approaches are recommended [3]. They have shown superior effectiveness in decreasing pain and disability compared to usual care [27, 28]. In this regard, a combination of conservative (e.g. pharmacological, physical, psychological) interventions are applied by specialists of diverse professions [16, 27, 29]. Building on this holistic treatment approach and to further improve the effectiveness, we have designed a novel multimodal exercise intervention (MultiMove) for CLBP patients. MultiMove addresses the described impaired physical (e.g. dynamic postural control, trunk stability), cognitive (e.g. executive functions) and psychological (e.g. kinesiophobia) domains of CLBP, through the combination of three training components.

The basic component of MultiMove consists of a strength and flexibility training, as they have been shown to be the most effective exercise interventions for CLBP patients, so far [30, 31]. To moreover address the impaired dual task performance and the executive functions of CLBP patients, the second component of MultiMove is a motor-cognitive training according to the Life Kinetik® concept [32]. In healthy older adults, this kind of training has already been shown to decrease gait variability as well as reduce fear of falling [33, 34]. Because balance/stabilisation training has been shown to be advantageous for CLBP patients [30], the third component comprises a dynamic balance training using dance-therapeutic elements. Moreover, dance-therapeutic exercises inherently require a combination of motor and cognitive functions (e.g. to remember and execute several partial movements at the same time) and are further associated with social interaction [35, 36]. In combination with the beneficial effects of the motor-cognitive training, this social interaction might help to overcome the kinesiophobia. In line with this, it has been shown that dance interventions improve functional mobility, sensorimotor and endurance performance in healthy adults and elderly [37–39]. Besides, dance interventions might lower gait variability and improve gait speed [40, 41]. Additionally, it has been revealed that dancing is superior to conventional training interventions (involving mainly repetitive physical exercises) with regard to the training-induced brain plasticity in elderly people [42]. Due to these multiple benefits, dance-therapeutic interventions have already been effectively applied in the field of neurological rehabilitation [36, 43–46]. Therefore, extending the approach to the needs of CLBP patients seems promising.

With the combination of these three components, MultiMove is expected to contribute to an improvement in physical and physiological functions (e.g. Timed Up-and-Go [TUG] performance, gait variability, haemodynamic response in the prefrontal cortex [PFC]) as well as cognitive performance [aspects of executive functions]). A reduction in pain as well as an increase in quality of life are assumed to come along with those changes.

### Primary objective

1. To test the effect of MultiMove on CLBP patients’ physical function (TUG performance) relative to the conventional conservative therapy.

### Secondary objectives

1. To examine changes in the LBP specific disability (Oswestry Disability Index, ODI) and health-related quality of life (EQ-5D-5L) between groups and over time.
2. To evaluate the specific physical changes induced by MultiMove (gait variability, flexibility, functional exercise capacity and leg extensor muscle power).
3. To analyse if MultiMove induces changes in selected executive functions (inhibitory control, cognitive flexibility) and/or in the haemodynamic response in the PFC during dual task standing and walking in CLBP patients.
4. To determine the potential long-term psychosocial effects (Kinesiophobia, pain coping strategies) provoked by MultiMove.

## Methods

### Study design and setting

**Fig. 1:**
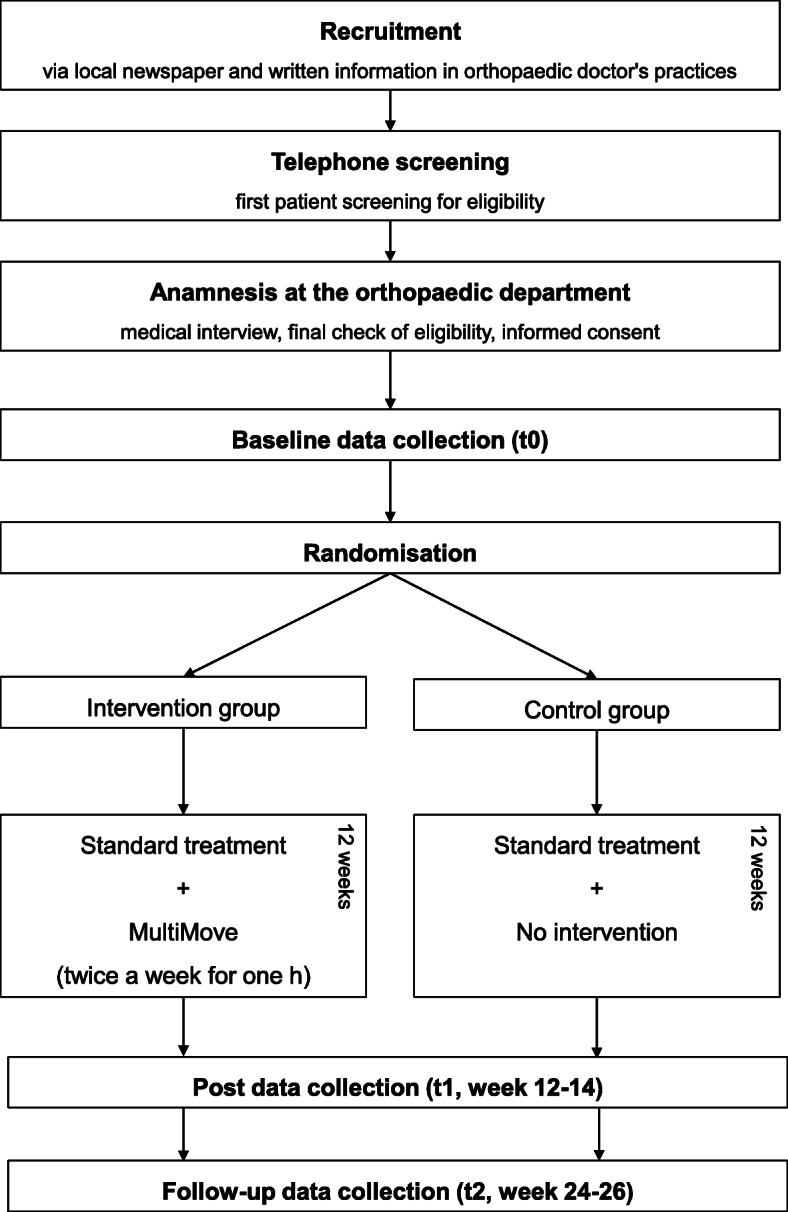
Study schedule of enrolment, intervention and assessments

The current study is designed as a prospective, two-arm randomized, controlled, superiority clinical trial. To assess the effect of MultiMove on the measures presented above in CLBP patients, subjects will be allocated to an intervention group (IG) or a control group (CG) using block randomization (1:1 allocation) [47]. The primary and secondary outcomes will be assessed at three time points (see Fig. 1, a detailed overview of the outcome measures can be found in Table 3).

The first measurements will be conducted directly before intervention onset (baseline), the second directly after the intervention (post, week 12–14, t1), and the third after additional 12 weeks (follow-up, week 24–26, t2). All investigators will be blinded to group allocation. Data collection will be performed at the Otto von Guericke University (OvGU) Magdeburg (Germany). The protocol is in line with the principles of the Declaration of Helsinki and was approved by the institutional review board (IRB) of the OvGU (registration number: 182/18).

### Study population and eligibility criteria

MultiMove is designed for outpatients with CLBP associated with degenerative diseases of the lumbar spine and aged ≥50. They will be recruited by the orthopaedic department of the Klinikum Magdeburg gGmbH in collaboration with local orthopaedist. Information will be provided in written form in doctor’s practices as well as personally in their consultations. In addition, the study will be advertised via local newspaper.

To be considered for the study, participants must comply all off the following inclusion criteria:

(i) age ≥ 50 years
(ii) an average low back pain over the last 4 weeks ≥4 on a numerical rating scale (NRS) from 0 - no pain to 10 - pain as bad as it could be
(iii) duration of LBP and/or neurogenic claudication ≥3 months
(iv) International Classification of Diseases 10th revision diagnosis code related to chronic back pain (ICD-10: M54 Dorsalgia; M48.0 Spinal stenosis; M54.5 Low back pain; M54.4 Lumbago with sciatica; M54.1 Radiculopathy; M41.5 Other secondary scoliosis; M43.1 Spondylolisthesis; M42.1 Adult osteochondrosis of spine; M51.2 Other specified intervertebral disc displacement; M47.8 other spondylosis; M53.2 Spinal instabilities)

Exclusion criteria are: (i) more than two prior operations of the spine, (ii) spinal fusion of more than three segments, (iii) any spinal operation within the last 6 months, (iv) dependence on a walking aid or inability to walk more than 300 m at a stretch, (v) reduction in strength of more than 25% according to Janda [48] (level 0–3), (vi) congenital spine deformities, (viii) any neurological, cardiovascular, psychological and musculoskeletal diseases that preclude the execution of the intervention and the measurements.

### Sample size

Sample size was calculated using G*Power (version 3.1.9.7.). Because previous studies have shown large effect sizes for changes in the TUG performance after single interventions (dancing [49], strength training [50]) as well as after multimodal interventions [51] in healthy older adults, a large effect size (f = 0.40) was assumed for the sample size calculation. Considering an α level of 0.05, a power of 0.95, two groups and two covariates (baseline scores and, when required, e.g. age, sex, etc.), the required total sample size for an analysis of covariance (ANCOVA) amounted 84 participants. With a hypothesized dropout rate of around 15%, a total sample size of 100 participants seems to be legitimated.

### Study interventions

Both groups (IG and CG) will receive the standard conservative therapy comprising physiotherapy and drug-based pain management according to their individual needs. Additionally, the participants of the IG will receive MultiMove twice a week on non-consecutive days over a period of 12 weeks. The duration of each session is set to 60 min (see Table 1) with a successive progression in the level of difficulty over time (see Table 2). Each session consists of motor-cognitive, dancing and strength or flexibility exercises with reference to the current recommendations for physical activity in older adults provided by the American College of Sports Medicine [52].

**Table 1:** Time slots for weekly training sessions (WTS) of 2 × 60 min in all phases

Training components	Phase 1–3	
	WTS 1* (Time)	WTS 2* (Time)
Life Kinetik® (including warm-up)	25 min	25 min
Dance	25 min	25 min
Strength	–	10 min
Flexibility	10 min	–

* ≥ 48 h rest between WTS

**Table 2:** Details of MultiMove with exercise examples

Training components	Exercise	Examples of exercise Phase 1	Examples of exercise Phase 2	Examples of exercise Phase 3
		Week 1–4 │ Foundational	Week 5–8 │ Intermediate	Week 9–12 │ Advanced
Life Kinetik®	cognitive-motor exercises	balancing while counting backwards	bouncing a ball while solving arithmetic tasks	ball juggling and reacting to external stimuli
Dance	choreographies focusing on different dance styles	Line dance, Irish dance	Pasodoble, Salsa, Cha-cha-cha	Tango, Salsa
Strength	core stability and strengthening	pelvic tilt, abdominal hollowing	curl up, bridge, bird dog	side plank, hip abductor wall squat
Flexibility	range of motion through static stretching of core and hip muscles	knee to chest, piriformis stretch	hamstring stretch, hip flexor stretch, seated flexion	warrior one pose, warrior two pose

The classes will start with the motor-cognitive component, which includes a warm-up phase with movements for all major muscle groups. The following motor-cognitive exercises are in line with a licensed concept (Life Kinetik®, [32]). Here, motor and cognitive tasks have to be performed simultaneously, requiring a redistribution of attentional resources. The exercises are designed in a way that they can hardly be executed without mistakes and the difficulty level will be adapted to the ability of the participants. During the following dance part, the participants will learn choreographies with increasing complexity over time, which will be adapted to their disability level. The choreographies will include different dance styles (e.g. Latin, Standard, Jazz) whereby each style focuses on a specific aspect, e.g. spatial orientation in Line dance or posture in Latin dance. To ensure that on the one hand different performance levels do not influence the dancing, but on the other hand, participants interact socially, the choreographies will be performed individually in a group setting and will be supplemented by dance formations. The final strength and flexibility training will focus on evidence-based exercises for the spine, back, abdominal and hip muscles [53].

The sessions will be supervised by two accredited instructors and will be conducted with a maximum of 15 participants. Instructors will keep an attendance list at each class. If participants are absent for two consecutive intervention sessions, they will be contacted by telephone. In order to improve attendance to MultiMove, participants of the IG will be asked to visit at least 80% of the classes. Moreover, participants will receive global information about the purpose and usefulness of the intervention at the first training session as well as specific explanations during the respective exercises. The individual perceived enjoyment of MultiMove will be assessed via the German version of the Physical Activity Enjoyment Scale once a week [54].

Additionally, instructors will keep an activity log in each class to maintain intervention fidelity. Activity log will include a check-list with essential components of the intervention protocol and will be reviewed by an independent assessor. If participants will not be able to perform the planned exercises, because of the physical requirements, small individually adjusted modifications will be made and protocolled. Furthermore, if participants should premature terminate out of MultiMove, reasons will be recorded (e.g. personal reasons, relocation, illness that prevent physical activity or death).

The participants of the CG will be examined in the same time period as the IG receiving only the standard therapy (physiotherapy and drug-based pain management according to their individual needs), with the possibility to attend MultiMove as well, but not before the follow-up data collection is completed.

Besides, both groups will be asked not to start any (further) specific training intervention or pain treatment during the study period. Their physical activity level will be monitored by a questionnaire.

### Outcome measures

A detailed description of the outcome measurements is following, whereby Table 3 presents an overview of the addressed constructs with the assessment methods and respective parameters. Moreover, the measurement time points are marked in Table 3.

**Table 3:** Constructs, assessment methods, parameters and data collection schedule

Constructs	Assessment methods	Parameters	Baseline (t0)	Post (t1)	Follow-up (t2)
Physical functions					
Mobility and function of the lower extremities	Timed Up-and-Go Test^1^	Time (s) to complete the task	X	X	X
Functional exercise capacity and dynamic postural control	Six-minute walk test with inertial measurement units (IMUs) and an electro-cardiogram	Walking distance (m) in 6 min, gait kinematics (e.g. minimum toe clearance, gait variability) and heart rate	X	X	X
Functional leg extensor muscle power	Five-repetition sit-to-stand test on a force plate	Time (s) and rate of force development	X	X	X
Flexibility	Instrumented trunk range of motion assessment	Active range of motion (°)	X	X	X
Subjective health state					
Back pain associated disability in daily life	Oswestry Disability Index	Total score (0–100%)	X	X	X
Health-related quality of life	EQ-5D-5L	Index value (−0.66 to 1) and subjective overall health state (0–100)	X	X	X
Motor-cognitive dual task performance					
Static and dynamic postural control during a cognitive challenge, haemodynamic response in the prefrontal cortex (PFC) and dual task costs	Single and dual task bipedal stance performance on a force plate (with and without closed eyes) as well as gait analysis with IMUs, functional near-infrared spectroscopy and finger oximeter	Deviation of the center of pressure (e.g. sway and velocity), gait kinematics (e.g. minimum toe clearance, gait variability, gait velocity), oxy- and deoxyhaemoglobin concentrations in the PFC, number of correct calculations and peripheral oxygenation	X	X	X
Executive functions					
Inhibitory control	Colour-Word-Interference Test	Time (s) and error rate	X	X	X
Cognitive flexibility	Trail Making Test	Time (s) and error rate	X	X	X
Psychosocial aspects					
Fear of movement	Tampa Scale of Kinesiophobia	Total score (11–44)	X	X	X
Pain coping strategies	Coping Strategies Questionnaire	Total score for each coping strategy (0–36)	X	X	X
Additional					
Sociodemographic data	Intake form	e.g. age, sex, education	X		
Physical characteristics	Intake form	e.g. height, weight	X	X	X
Current pain intensity	Numerical rating scale	Score (0–10)	X	X	X
Specific characteristic and treatment history of chronic pain	German Pain Questionnaire	Descriptive data, time period (months) of chronic pain, pain intensity (0–10) in the last 4 weeks	X	X	X
Level of everyday activity	Freiburger Questionnaire on Physical Activity	Time (h) of physical activity in a week	X	X	X
State fatigue	Fatigue subscale of the Profile of Mood States	Score (0–42)	X	X	X
Depression	Beck Depression Inventory II	Total score (0–63)	X	X	X

^1^Primary outcome measure; all instruments will be presented in German

### Primary outcome measure

#### Timed Up-and-Go Test

The TUG Test is commonly used in geriatric settings to assess function of the lower extremities, mobility and fall risk [55]. The patient will sit on a standard arm chair with his back and arms resting on it. A line will be marked on the floor 3 m away from the chair. At the command “go” the patient has to get up (without using the arms), walk as quickly and stable as possible to the marked line, turn 180°, return to the chair and get back into the starting position [55, 56]. To ensure, that the test procedure has been understood, the procedure can be tried once before the actual test. The time needed from releasing the back from the backrest to touching it again will be measured during the test. The TUG will be performed two times, whereby the fastest trial will be considered. It has been shown, that the TUG test can be performed reliably [55, 57].

### Secondary outcome measures

#### Physical functions

##### Six-minute walk test

The Six-minute walk test (6MWT) is an objective measurement to assess the functional exercise capacity close to activities of daily living by measuring the total walking distance accomplished in 6 min [58]. During the 6MWT, the participants will walk back and forth on a 15 m long track as fast as they can for 6 min. Additionally to the standard protocol, gait kinematics (e.g. minimum toe clearance (MTC) [59], double step length (DSL), gait velocity [60] and their variability) will be recorded, using three inertial measurement units (IMUs, one at each foot and one at the sternum, MTw, Xsens Technologies B.V., Netherlands). The calculation of gait parameters will be in line with the protocol of Hamacher et al. [59]. Furthermore, the participants will wear a portable 3-channel electrocardiogram with a finger oximeter (SOMNOtouch™ NIBP; SOMNOmedics GMbH Germany) to assess heart rate and peripheral oxygenation as an objective measurement of exercise intensity. Additionally, the subjective level of exhaustion [61] as well as the current pain situation on a NRS from 0 (not fatigued at all / no pain) to 10 (total fatigue & exhaustion / pain as bad as it could be) will be enquired before and after the 6MWT.

##### Five-repetition sit-to-stand test

The leg extensor muscle power will be assessed with the five-repetition sit-to-stand test (FRSTST) [62, 63] performed on a force plate (Type 9260AA, Kistler Group, Winterthur, Switzerland; sampling frequency: 1000 Hz). The participants will sit on a standard chair without armrest in front of a force plate. They will be asked to fold their arms across their chests, place their feet on the plate and to stand up and sit down as fast as possible five times in a row [62, 63]. During the performance, the investigator will ensure that the participants getting up fully upright and touch the surface of the seat completely. Participants are allowed to practice the process two times. The total time, from the starting signal to the fifth time seating, and the force-time curves, from which rate of force development can be derived, will be measured.

##### Instrumented trunk range of motion assessment

A sensor-based measuring device (mobbe®med, SportMed A.G. SA, Luxembourg) will be used to assess active trunk range of motion. Specifically, the participants will be asked to perform extension-flexion, lateral flexion (left and right) and rotation (left and right) of the spine. Thereby, the range of motion as well as the subjective pain sensation rated on a NRS (0–10) will be recorded.

#### Subjective health state

##### The Oswestry disability index

The ODI is a condition-specific, self-administered questionnaire for spinal disorders [64, 65]. It consists of 10 items, scored on a 6-point scale (0 to 5). One item assesses the extent of back pain, while the other nine ask for the difficulties in different activities of daily life because of the pain: personal care, lifting, walking, sitting, standing, sleeping, sex life, social life and travelling [66]. All items refer to the current (“today”) pain situation. The total score is multiplied by 2 and is presented as a percentage, whereby a higher score represents a higher level of disability [66]. In this study, the reliable and valid German version of the ODI will be used [66].

##### EQ-5D-5L

The EQ-5D-5L has been developed by the EuroQol Group and is an international, standardized, short questionnaire to assess generic health-related quality of life [67]. In the current study the German self-complete paper version will be used, which consists of two parts. The first part includes five items (health dimensions: mobility, self-care, usual activities, pain/discomfort and anxiety/depression). Here, each dimension can be scored on a 5-point scale (severity levels) corresponding to the problems the respondent has in this area (0 = no problem, 5 = extreme problems). The second part consists of a visual analogue scale (VAS) ranging from 0 to 100 (worst to best health the respondent can imagine), on which participants should indicate their current subjective overall health state. To calculate a EQ-5D-5L summary index score a country specific value set, which weights each level in each dimension specifically, will be used [68].

#### Motor-cognitive dual task performance

##### Single and dual task postural control performance with functional near-infrared spectroscopy and inertial measurement units

A portable functional near-infrared spectroscopy (fNIRS/NIRSport, NIRx Medical Technologies, NY, USA) and IMUs (described above) will be applied while performing postural control tasks with and without a cognitive task. Due to the fNIRS system requirements, the testing protocol has to alternate constantly between baseline (standing position with eyes open) and one of four action tasks. First two dynamic postural control tasks are conducted in random order: single task walking (STW) or dual task walking (DTW). Subsequently, two static postural control tasks - dual task standing (DTS) or closed eyes standing (CES) - are executed in a random order. For the dynamic conditions, the participants will have to walk a 15 m track back and forth with their individual comfort velocity. During static conditions, participants will stand as stable as possible on a force plate (Type 9260AA, Kistler Group, Winterthur, Switzerland; sampling frequency: 100 Hz) in an upright bipedal position with arms akimbo, looking straight ahead. The positions of the feet will be measured and noted, to ensure reliable measurements. In the dual task conditions, the participants will count backwards in steps of three from a pre-defined 3-digit number (between 300 and 400) while walking (DTW) or standing (DTS). Here, participants will be instructed to pay equal attention to both tasks. During the CES participants will be asked to close their eyes while continuing the stable stand.

Each action part will be assessed over a total of 2 min (split in four times 30 s) and the baseline over 2:45 min (five times 33 s). Hence, the total measuring time will be 4:45 min per task. In order to familiarize the participants with each task, a short version (10 s:10 s) will be performed once before actual testing. During all four tasks, the haemodynamic response in the PFC (relative oxy- and deoxyhaemoglobin concentrations) will be measured by a fNIRS system. Data recording, processing and analyses will be in line with the recently published guidelines for fNIRS in posture and gait research [69]. Additionally, gait kinematics (e.g. MTC, DSL, gait velocity and their variability) will be recorded with IMUs during the dynamic postural exercises. During the static tasks the deviation of the centre of pressure (e.g. sway and velocity) will be assessed based on the force plate data. The error rate and number of correct calculations of the cognitive task will be captured with a voice recorder. Furthermore, the participants will wear the SOMNOtouch (described above) to check for confounders in the haemodynamic response [70]. Here the outcome parameters are: heart rate, heart rate variability indices and peripheral finger oxygenation.

#### Executive functions

##### Colour-Word-Interference test

The Colour-Word-Interference Test (based on the Stroop effect [71]) is a frequently used neuropsychological test to assess inhibitory control [72, 73]. Here, the participants have to read out loud and as fast as possible nine tables. These tables consist of three different consecutive tasks that are presented three times: (i) read colour names (printed in black ink), (ii) name the colour of colour bars, (iii) name ink colour of colour names (printed colour never matches the written colour name) [71, 73]. The (iii) task is the incongruent condition requiring to inhibit the more automated task (e.g. reading the colour name), which is called the Stroop effect [71, 73]. The required time is stopped individually for each table and the corrected and uncorrected errors are determined for condition (iii). Additionally, the time difference between (ii) and (iii) is calculated [74]. In this study the German paper-pencil version of the Colour-Word-Interference Test will be used (Farbe-Wort-Interferenztest, [75]).

##### Trail Making Test (part A and B)

The Trail Making Test (TMT) is a neuropsychological test of visual search, processing speed and cognitive flexibility [76]. It consists of two tasks A and B, each requiring to connect 25 consecutive targets as quickly and accurately as possible. In part A, numbers (1–25) presented randomly on a piece of paper have to be connected in ascending order as fast as possible. Part B requires mental shifting between presented numbers (1–13) and letters (A-L). They have to be connected in alternating order (1, A, 2, B…). The processing time for each part will be recorded.

#### Psychosocial aspects

##### Tampa Scale of Kinesiophobia

The Tampa Scale of Kinesiophobia - German version (TSK-GV) is a valid and reliable 11-item self-report questionnaire measuring the fear of movement and reinjury [77]. Participants are asked to rate each item between one (strongly disagree) and four (strongly agree). Hence, a higher overall score represents a stronger individual’s fear of movement [77].

##### Coping Strategies Questionnaire

The Coping Strategies Questionnaire is a reliable measure for pain coping techniques [78]. In this study, the German version will be used (CSQ-D) [79]. The CSQ-D comprises 50 items in total. The first 48 items describe different cognitive or behavioural coping techniques (cognitive: diverting attention, reinterpreting pain sensations, coping self-statements, ignoring pain sensations, praying or hoping, catastrophizing; behavioural: increasing activity level, increasing pain behaviours). Participants are asked to rate how frequently they use each of the described coping techniques on a 7-point scale (0 = never, 6 = always). Additionally, two items assess their self-reported overall effectiveness of their strategies. To evaluate the CSQ-D, a score is calculated for each coping technique ranging from 0 to 36 [79].

### Additional measurements

Additionally, demographic (e.g. age, sex, education), physical characteristics (e.g. height, weight) and the current pain intensity on a NRS (0 - no pain to 10 - pain as bad as it could be) will be assessed. Moreover, information which could possibly influence the treatment outcome will be recorded. Hence, the specific characteristics and treatment history of chronic pain will be evaluated with parts of the German Pain Questionnaire [80, 81]. The level of everyday physical activity will be assessed with the Freiburger Questionnaire on Physical Activity, collecting self-assessed data to the amount of leisure and work related activity [82, 83]. The level of state fatigue and depressive symptoms at the measurement days will be estimated trough the fatigue subscale of the Profile of Mood States (POMS-F) [84] and the Beck Depression Inventory II (BDI-II) [85].

The participants will receive a medical check-up at the end of the study period at the same orthopaedic department where the initial check-up takes place, to recognize beneficial and adverse events related to the study intervention. Any other harms, occurring during the study intervention, will be protocolled by the accredited instructors.

### Study procedure

Interested subjects who will become aware of the study through the provided information (described above) can contact the research assistance of the Klinikum Magdeburg gGmbH via telephone or e-mail (see Fig. 1). Subsequently, a first eligibility check will be performed by telephone. An enrolment list will be created and constantly updated by the research assistance. Potentially eligible patients will be referred to the ambulatory health care centre of the Klinikum Magdeburg gGmbH to be informed in detail and to obtain written consent. Additionally, a medical interview will be conducted, to assess the pain characteristics and medical eligibility criteria. Finally, patients will be given a questionnaire catalogue (demographic and physical characteristics, German Pain Questionnaire, the Freiburger Questionnaire on Physical Activity, TSK-GV and CSQ-D) advising that this catalogue should be completed shortly before the pre-assessment. Subsequently, all necessary information will be forwarded to the OvGU and the participants will be invited to the pre-measurement. Two days before, they will be reminded via telephone to bring along the completed questionnaire.

Each of the measurement appointments will take about 2.5 h. At the beginning participants will complete a set of questionnaires, including the ODI, EQ-5D-5L, POMS-F and BDI-II. Afterwards, the Colour-Word-Interference Test will be performed, followed by the flexibility assessment, the TUG, the FRSTS, the dual tasks with IMUs and fNIRS, the TMTA and B and finally the 6MWT. The group allocation will be concealed until the baseline data collection is completed. For this purpose, the enrolment list will be transferred to the OvGU, where BK will perform the group assignment based on the allocation sequence generated by MB. All participants who fulfil the inclusion criteria and give consent, will be randomly assigned to either the IG or CG group with a 1:1 allocation. Block randomization with a block size of ten will be used and the allocation is generated via a random number table. Participants will be informed by phone about their group assignment. To promote participant retention and completion of all measurements, participants will receive a financial compensation at the follow-up data collection. Moreover, the results of this study will be published in peer-reviewed journals and will be communicated with the patients and partners.

### Data analysis and management

Data will be analysed using IBM SPSS 26.0 (SPSS Inc., Chicago, IL). At first, a descriptive analysis will be conducted to identify outliers, distributions and to display the baseline characteristics of both groups (means, proportions, standard deviations). To evaluate the effects of the intervention, data of all patients will be processed, regardless of whether they fully received or dropped out of the intervention (intention-to-treat-analysis). Thereby, data will be analysed until the date of drop out. As a secondary analysis, only the data from the participants who participated at least 80% of the intervention will be included in a per-protocol analysis. Missing data will be imputed using multiple imputation, under the assumption that data are missing at random. Statistical significance level will be set at *p* ≤ 0.05 (1-sided).

In order to analyse differences between the groups at the time points post and follow-up, two ANCOVAs with baseline adjustments will be undertaken (covariates: baseline values and, when required, e.g. age, sex etc.) for all outcome measures. Subsequently, alpha-adjustments will be undertaken. In case the assumptions for the ANCOVA will be violated, mixed models will be used. To further elaborate the outcomes and their interactions, additional parametric or nonparametric statistical analyses will be applied. Exploratory mediation analysis will be performed supplementary.

To preserve the confidentiality, all participants will receive a specific code with which the data will be labelled, while the participants contact data will remain under lock. The collected data will be stored digitally and can only be accessed by the study group. Therefore, the data will be transmitted by one person and checked for correctness by another (transmission errors and plausibility). In this study, no data monitoring committee and interim analysis will be established, because of the minimal risks associated with the intervention. Overall, the data management will be in line with of the European General Data Protection Regulation.

## Discussion

To the best of our knowledge, MultiMove is the first study combining motor-cognitive with dance-therapeutic as well as strength and flexibility training to address the multifaceted impairments of CLBP. First of all, it is expected that the combination of these three components will stimulate the participants in moving their spine constantly and therefore counteract the fear of moving. The group-setting will additionally provide the opportunity to experience mutual support and encouragement from peers in the engagement for physical activities [38].

Furthermore, the hypothesised advantage of MultiMove for CLBP patients is seen in the intertwining effects of the three training components. The basic component with its specific strengthening and flexibility exercises targets the whole lumbar spine including the lumbar multifidus and transversus abdominus muscles, which are known to promote the pain situation in CLPB patients [86]. The assumed enhanced lumbar stability could counteract the degeneration processes of the vertebrae and joint structures and therefore build the foundation for functional performances required during everyday activities and addressed in the motor-cognitive and dance-therapeutic component of MultiMove. Moreover, most of the movements performed in daily life are accompanied by a second task (e.g. walking and talking). This aspect will be targeted during the motor-cognitive training, where the patients will be confronted with constantly novel exercises requiring interactions between cognitive and physical performance. This ability, learned in a playful setting, might be especially valuable in enhancing the dual task performance of the patients. The following dance-therapeutic intervention stresses the mentioned aspects, as it addresses the trunk range of motion and incorporates cognitive tasks. Although, it has been shown that a dancing program is suitable to improve the motor-cognitive dual task capability in elderly [41], this study did not investigate brain activity during dual task walking. Consequently, a major advantage of this study can be seen in the methodological approach. Due to the combination of neuropsychological tests, gait analysis and fNIRS, a detailed elaboration of the respective influences on the motor-cognitive performance will be possible. These results will contribute to a better understanding which of these entities have to be addressed to reduce the impairments of CLBP patients. Furthermore, the randomized controlled design with the additional follow-up measurement will enable high level evidence on the long-term effects of MultiMove. Finally, due to the aspect that MultiMove does not need special requirements, it could be delivered on low cost in all areas, where it is needed.

One limitation in the application of MultiMove is that the participants must be physically able to participate actively in the intervention over 60 min. Hence, CLBP patients with severe physical impairments cannot be included.

The presented study is only the first stage of a three staged project. In the second stage, it is intended to test an adapted multimodal treatment during inpatient rehabilitation and in the third stage, the programme will be integrated into the medical prevention. The overall objective is to establish the multimodal treatment into cross-sectoral care to sustainably improve the situation of CLBP patients along the entire medical supply chain.

## Data Availability

Not applicable.

## Abbreviations

BDI-II: Beck Depression Inventory II
CES: Closed eyes standing
CG: Control group
CLBP: Chronic low back pain
CSQ-D: Coping Strategies Questionnaire – German Version
DSL: Double step length
DTS: Dual task standing
DTW: Dual task walking
FRSTST: Five-repetition sit-to-stand test
fNIRS: Functional near-infrared spectroscopy
ICD-10: International Classification of Diseases 10th revision
IG: Intervention group
IMUs: Inertial measurement units
IRB: Institutional review board
LBP: Low back pain
MTC: Minimum toe clearance
MultiMove: Multimodal intervention
NRS: Numerical rating scale
ODI: Oswestry Disability Index
OvGU: Otto von Guericke University
POMS-F: Profile of Mood States- fatigue subscale
PFC: Prefrontal cortex
STW: Single task walking
TMT: Trail Making Test
TSK-GV: Tampa Scale of Kinesiophobia – German Version
TUG: Timed Up-and-Go
6MWT: 6-min walk test
